# Protein Expression of Angiotensin-Converting Enzyme 2 (ACE2) is Upregulated in Brains with Alzheimer’s Disease

**DOI:** 10.3390/ijms22041687

**Published:** 2021-02-08

**Authors:** Qiyue Ding, Nataliia V. Shults, Sergiy G. Gychka, Brent T. Harris, Yuichiro J. Suzuki

**Affiliations:** 1 Department of Pharmacology and Physiology, Georgetown University Medical Center, Washington, DC 20007, USA; qd27@georgetown.edu (Q.D.); ns1015@georgetown.edu (N.V.S.); 2 Department of Pathological Anatomy N2, Bogomolets National Medical University, 01601 Kiev, Ukraine; gychka59@gmail.com; 3 Departments of Neurology and Pathology, Georgetown University Medical Center, Washington, DC 20007, USA; bth@georgetown.edu

**Keywords:** Alzheimer’s disease, ACE2, angiotensin-converting enzyme 2, brain, coronavirus, COVID-19, oxidative stress

## Abstract

Alzheimer’s disease is a chronic neurodegenerative disorder and represents the main cause of dementia globally. Currently, the world is suffering from the coronavirus disease 2019 (COVID-19) pandemic, caused by severe acute respiratory syndrome coronavirus 2 (SARS-CoV-2), a virus that uses angiotensin-converting enzyme 2 (ACE2) as a receptor to enter the host cells. In COVID-19, neurological manifestations have been reported to occur. The present study demonstrates that the protein expression level of ACE2 is upregulated in the brain of patients with Alzheimer’s disease. The increased ACE2 expression is not age-dependent, suggesting the direct relationship between Alzheimer’s disease and ACE2 expression. Oxidative stress has been implicated in the pathogenesis of Alzheimer’s disease, and brains with the disease examined in this study also exhibited higher carbonylated proteins, as well as an increased thiol oxidation state of peroxiredoxin 6 (Prx6). A moderate positive correlation was found between the increased ACE2 protein expression and oxidative stress in brains with Alzheimer’s disease. In summary, the present study reveals the relationships between Alzheimer’s disease and ACE2, the receptor for SARS-CoV-2. These results suggest the importance of carefully monitoring patients with both Alzheimer’s disease and COVID-19 in order to identify higher viral loads in the brain and long-term adverse neurological consequences.

## 1. Introduction

Alzheimer’s disease is a chronic neurodegenerative disorder and the main cause of dementia around the world, affecting a large number of aging populations with an estimated prevalence of 10–30% in the population aged >65 years [1,2]. In the United States alone, an estimated 5 million people have Alzheimer’s disease, which is the fifth leading cause of death among older adults in the country. However, little is known about the cause of Alzheimer’s disease and no curative treatments are presently available [3,4]. Oxidative stress and the production of reactive oxygen species (ROS) [5,6] have been implicated in Alzheimer’s disease [7,8,9,10], yet clinical trials on antioxidants have not shown promise as effective treatments [11,12]. Thus, a further understanding of the role of oxidative stress in Alzheimer’s disease is needed.

Coronaviruses are positive-sense single-stranded RNA viruses that often cause the common cold [13,14]. Some coronaviruses, however, can be lethal, as the world witnessed in the 2020 pandemic. Severe acute respiratory syndrome coronavirus 2 (SARS-CoV-2) caused the coronavirus disease 2019 (COVID-19) [15,16]. Thus far, over 90 million people have been infected with SARS-CoV-2 worldwide, causing serious health, economical, and sociological problems. SARS-CoV-2 uses angiotensin-converting enzyme 2 (ACE2) as a receptor to enter the host cells [17,18]. It has been noted that elderly individuals, especially those with cardiovascular disease, are highly susceptible to developing severe COVID-19 conditions [16,19,20].

While the current focus is on treating the lung and cardiovascular aspects of COVID-19 to reduce mortality, it has been reported that neurological manifestations occurred in 36% of COVID-19 patients [21], indicating that this disease may exert long-term neurological consequences. Thus, understanding the relationships between COVID-19 and the brain is important. The present study monitored the expression level of ACE2, the host cell receptor for SARS-CoV-2, and found that ACE2 protein is upregulated in the brains of patients with Alzheimer’s disease.

## 2. Results

### 2.1. ACE2 Protein Expression

**Figure 1:**
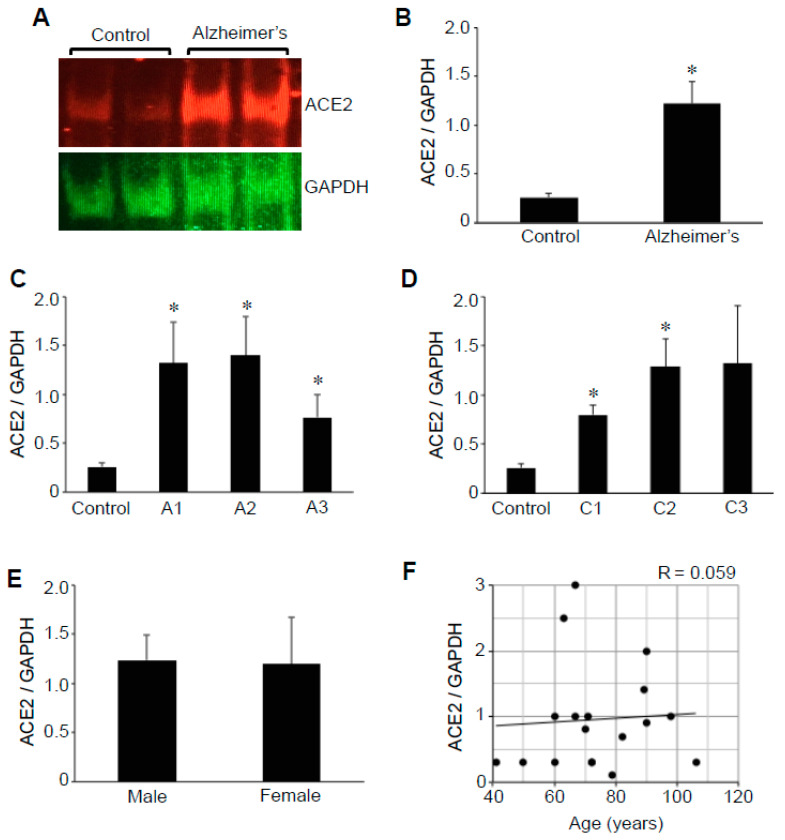
The ACE2 protein expression is upregulated in the brains of patients with Alzheimer’s disease. Brain (hippocampus) homogenates from control subjects and patients with Alzheimer’s disease were subjected to Western blotting using antibodies against ACE2 and GAPDH. (**A**) Representative results. (**B**) The bar graph represents means ± SEM of the ratio of ACE2 to GAPDH (N = 13 for Alzheimer’s and 5 for control). (**C**) The bar graph represents means ± SEM of the ratio of ACE2 to GAPDH for various Aβ plaque scores (denoted by the letter A). N = 5 for control, 5 for A1, 5 for A2, and 3 for A3. (**D**) The bar graph represents means ± SEM of the ratio of ACE2 to GAPDH for various neuritic plaque scores (denoted by the letter C). N = 5 for control, 2 for C1, 7 for C2, and 4 for C3. The symbol * denotes that the value is significantly different from the control value at *p* < 0.05. (**E**) The bar graph represents means ± SEM of the ratio of ACE2 to GAPDH (N = 8 for male and 5 for female). No significant difference was found between males and females. (**F**) The scattered graph represents age vs. ACE2 expression (N = 18). Pearson correlation coefficient R = 0.059 (weak positive correlation).

The hippocampus is the major structure in the brain affected by Alzheimer’s disease [22,23]. We performed Western blotting to determine the protein expression levels of ACE2 in hippocampal brain tissues from patients with Alzheimer’s and compared them with those of control subjects. The ACE2 protein expression was found to be higher in brains with Alzheimer’s compared with those of controls (Figure 1A). Quantifications of the ratio of ACE2 to glyceraldehyde 3-phosphate dehydrogenase (GAPDH) values revealed that the mean difference was 4.7-fold and statistically significant (Figure 1B). The mean ACE2/GAPDH ratio value for 13 patients with Alzheimer’s disease was 1.22 ± 0.2, while those of five control subjects were highly consistent with a mean of 0.26 ± 0.04. The expression levels of GAPDH were comparable between the control group (21,554 ± 1021 densitometry units) and the Alzheimer’s group (21,315 ± 1471 densitometry units).

The severity of Alzheimer’s as assessed by ABC grading [24] in the patients examined in this study was mostly in the intermediate to high range with 61.5% of patient samples exhibiting Aβ plaque scores of 2 or 3 and 84.6% exhibiting neuritic plaque scores of 2 or 3. The analysis of ACE2 protein expression in relation to Aβ plaque scores (Figure 1C) or neuritic plaque scores (Figure 1D) determined that the ACE2 expression levels are not related to the severity of the disease. Thus, the significant upregulation of ACE2 occurs even in patients with low-severity Alzheimer’s disease. Further, among the patients with Alzheimer’s disease, no gender difference in ACE2 levels was noted (Figure 1E). In the present study, the age of patients with Alzheimer’s disease ranged from 60 to 106 (mean = 78.8 ± 4.2 years) and that of control subjects ranged from 41 to 79 (mean = 60.4 ± 7.8 years), although our analysis detected no age-dependence in the ACE2 protein expression (Figure 1F).

**Figure 2:**
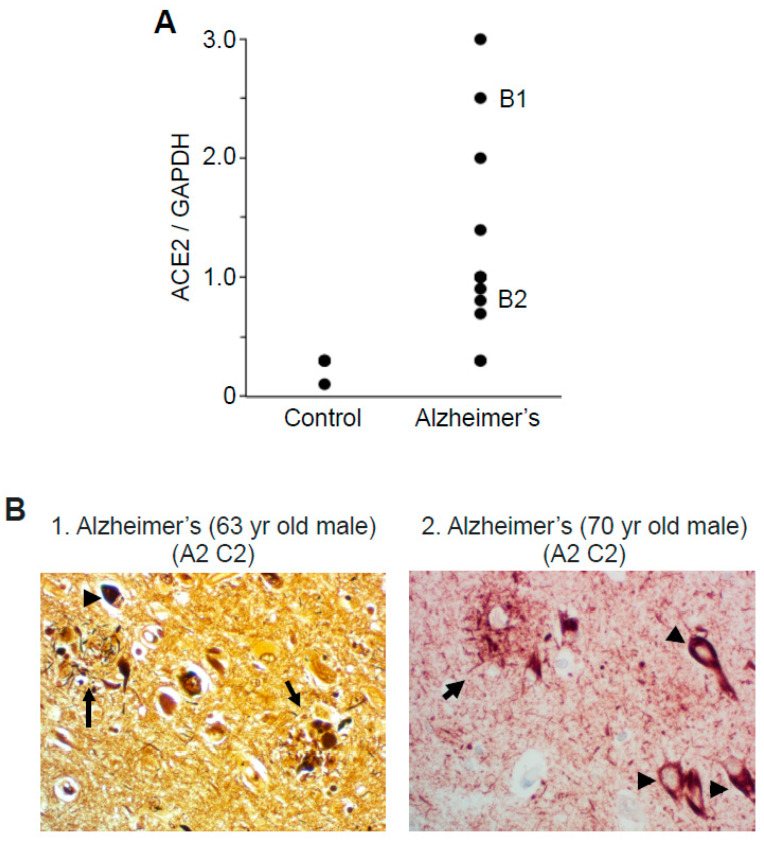
Variations within groups for results of ACE2 expression and histological evaluations of patients with Alzheimer’s disease. (**A**) Brain (hippocampus) homogenates from control subjects and patients with Alzheimer’s disease were investigated by Western blotting using antibodies against ACE2 and GAPDH. The dot graph represents values of the ratio of ACE2 to GAPDH for each control subject and patients with Alzheimer’s disease. Panel numbers of patients whose histology results are shown in Panel B are indicated in the graph. (**B**) Formalin-fixed, paraffin-embedded hippocampus tissues from patients with Alzheimer’s disease were cut and subjected to Bielschowsky silver staining (B1) or PHF-1/p-Tau immunohistochemistry (B2). Age, gender, Aβ plaque score (denoted by the letter A), and neuritic plaque score (denoted by the letter C) are noted for each slide. Brain parenchyma contains lesions such as neuritic plaques characterized by a dark amyloid core (arrows) and neurofibrillary tangles (arrowheads).

The ACE2/GAPDH ratio values obtained from 13 patients with Alzheimer’s disease ranged from 0.3 to 3 (mean = 1.2 ± 0.2), while those of control subjects were highly consistent with a mean of 0.26 ± 0.04 (Figure 2A). Silver staining images shown in Figure 2 (Panel B1) of our formalin-fixed paraffin-embedded tissues from patients with Alzheimer’s showed amyloid and neuritic plaques, as well as the neurofibrillary tangles [25,26]. Further, hematoxylin and eosin staining detected the amyloid plaques [27,28,29], and immunohistochemistry using the Tau AT8 antibody demonstrated the expression of phosphorylated Tau at serine 202 and threonine 205 [30] in our hippocampal tissue samples from patients with Alzheimer’s disease (Figure 2, Panel B2).

### 2.2. Protein Carbonylation

**Figure 3:**
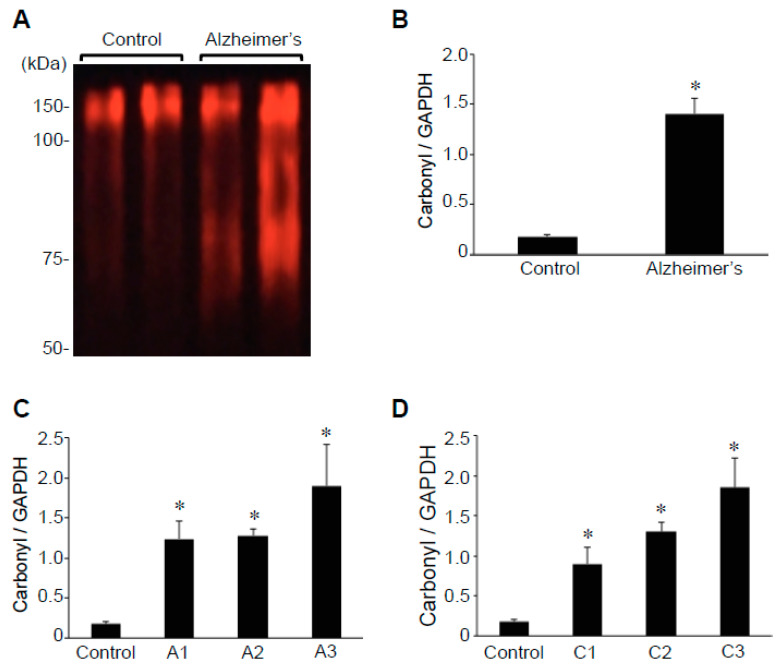
Protein carbonylation is upregulated in the brains of patients with Alzheimer’s disease. Brain (hippocampus) homogenates from control subjects and patients with Alzheimer’s were studied using OxyBlot. (**A**) Representative results. (**B**) The bar graph represents means ± SEM of the ratio of carbonylated proteins to GAPDH (N = 13 for Alzheimer’s and 5 for control). (**C**) The bar graph represents means ± SEM of the ratio of carbonylated proteins to GAPDH for various Aβ plaque scores (denoted by the letter A). N = 5 for control, 5 for A1, 5 for A2, and 3 for A3. (**D**) The bar graph represents means ± SEM of the ratio of carbonylated proteins to GAPDH for various neuritic plaque scores (denoted by the letter C). N = 5 for control, 2 for C1, 7 for C2, and 4 for C3. The symbol * denotes that the value is significantly different from the control value at *p* < 0.05.

Oxidative stress has been shown to occur in the brains of individuals with Alzheimer’s disease [7,8,9,10]. Consistently, protein carbonylation of various hippocampal proteins, as monitored by the 2,4-dinitrophenylhydrazine (DNPH) derivatization of carbonylated proteins using the OxyBlot Kit, showed higher carbonylated proteins in brains with Alzheimer’s compared with those of controls (Figure 3A). Quantifications of the results revealed that the protein carbonyl content is 7.8-fold higher in patients with Alzheimer’s disease compared with controls, with the difference statistically significant (Figure 3B). The carbonyl content-to-GAPDH ratio values ranged from 0.7 to 2.9 (mean = 1.4 ± 0.15) in brains with Alzheimer’s disease, while those of controls had a range of 0.1 to 0.2 (mean = 0.2 ± 0.02). Our analysis of Aβ plaque scores (Figure 3C) or neuritic plaque scores (Figure 3D) in relation to hippocampal brain protein carbonyl content may have shown a tendency to be dependent on the severity of Alzheimer’s disease, but no significant differences were noted among patient brain tissues. The protein carbonyl content was found to be consistently higher with statistical significance between controls and patients with Alzheimer’s of any degree of severity.

### 2.3. Protein Redox State Monitoring Kit Plus Analysis of Peroxiredoxin (Prx6)

The -*SulfoBiotics*- Protein Redox State Monitoring Kit Plus allows for the assessment of the thiol redox status of a given protein by using 15 kDa Protein-SHifters, which are added to the reduced cysteine sulfhydryl groups followed by immunological detection of the proteins of interest [31]. In the case of human Prx6 with both cysteine residues oxidized, no Protein-SHifter binds; thus, the protein migrates at 25 kDa (i.e., the molecular weight of Prx6). If one cysteine is reduced, then one 15 kDa Protein-SHifter binds to this cysteine, forming a 40 kDa complex, shifting the Prx6 band. If both cysteine residues are reduced, two Protein-SHifters bind to the protein, thus allowing for a total of 30 kDa shift in the 25 kDa Prx6, forming a 55 kDa species. We have previously found that, in untreated cultured human smooth muscle cells, Prx6 molecules are predominant in the reduced state, exhibiting a 55 kDa band in the -*SulfoBiotics*- Protein Redox State Monitoring analysis [31]. The treatment of cells with hydrogen peroxide resulted in the conversion of the 55 kDa band into mostly 40 kDa, rather than 25 kDa, suggesting that only one of the two cysteines in human Prx6 protein is susceptible for this oxidation [31]. A series of experiments determined that the shift of 55 to 40 kDa species is due to the oxidation of the catalytic cysteine 47 [31].

**Figure 4:**
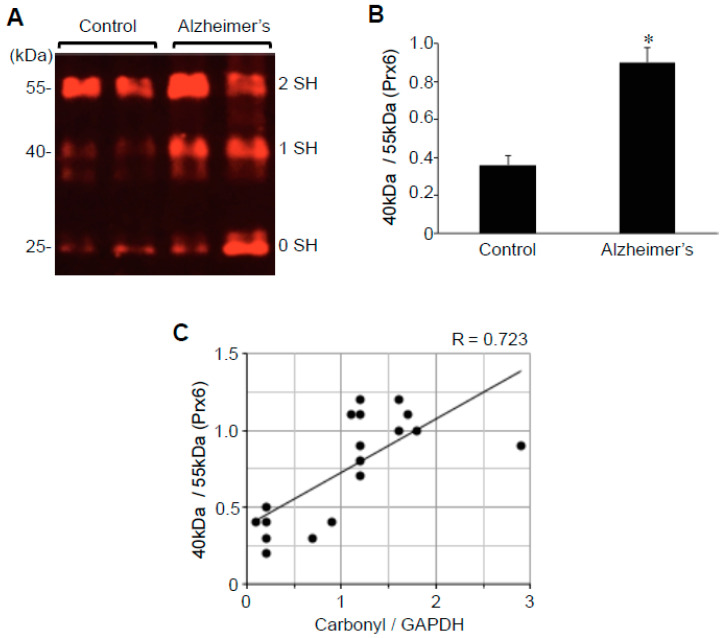
Cysteine oxidation of Prx6 is upregulated in the brains of patients with Alzheimer’s disease. Brain (hippocampus) homogenates from control subjects and patients with Alzheimer’s were investigated for cysteine oxidation of Prx6 using -*SulfoBiotics*- Protein Redox State Monitoring Kit Plus. A free protein thiol group was labeled with the Protein-SHifter Plus that contains maleimide with a high affinity toward reduced sulfhydryl groups. Each Protein-SHifter causes a 15 kDa shift. After electrophoresis, the Protein-SHifter Plus moiety was eliminated by exposure of the gel to ultraviolet light that increases the efficiency of Western blotting and allows for detection of specific proteins in biological samples. In the case of human Prx6, the protein with fully oxidized cysteine residues migrates at 25 kDa. The protein with one reduced cysteine binds to a Protein-SHifter and migrates at 40 kDa, and the protein with two reduced cysteine residues migrates at 55 kDa. Our previous study determined that the 40 kDa band depicts Prx6, in which the catalytic cysteine (Cys47) is oxidized [31]. (**A**) Representative Western blotting results using the Prx6 antibody showing 25 kDa (0 reduced sulfhydryl), 40 kDa (1 reduced sulfhydryl), and 55 kDa (2 reduced sulfhydryl) bands. (**B**) The bar graph represents means ± SEM of the ratio of the 40 kDa Prx6 band to 55 kDa Prx6 band (N = 13 for Alzheimer’s and 5 for control). The symbol * denotes that the value is significantly different from the control value at *p* < 0.05. (**C**) The scattered graph represents protein carbonylation vs. Prx6 thiol oxidation (N = 18). Pearson correlation coefficient R = 0.723 (positive correlation).

Using this system, we monitored protein thiol status in hippocampal brains of patients with Alzheimer’s and controls. Similar to the situation that can be produced by the treatment of cultured cells with hydrogen peroxide [31], we found that a large portion of Prx6 protein molecules in brains with Alzheimer’s exist as the 40 kDa species that corresponds to the addition of one Protein-SHifter (Figure 4A). Densitometry analysis determined that the ratio of 40 to 55 kDa Prx6 species is 2.5-fold higher in the brains of patients with Alzheimer’s compared with those of controls (Figure 4B), suggesting that catalytic cysteine is preferentially oxidized in the brains of the former. By contrast, no significant differences were noted for either the 25 to 55 kDa or 25 to 40 kDa ratio, indicating that noncatalytic cysteine is not consistently oxidized in the brains with Alzheimer’s disease. The range of the ratio of 40 to 55 kDa Prx6 species in patients with Alzheimer’s disease was from 0.3 to 1.2 (mean = 0.9 ± 0.08), while that of controls was from 0.2 to 0.5 (mean = 0.36 ± 0.05). Prx6 thiol oxidation as measured by the ratio of 40 to 55 kDa species was found to correlate with protein carbonylation (Figure 4C).

The analysis of the Aβ plaque or neuritic plaque scores in relation to the ratio of 40 to 55 kDa Prx6 species showed no statistically significant differences noted among patients’ brain tissues, while the Prx6 thiol oxidation was consistently higher with statistical significance between controls and patients with Alzheimer’s of any degree of severity.

### 2.4. Prx6 Protein Expression

**Figure 5:**
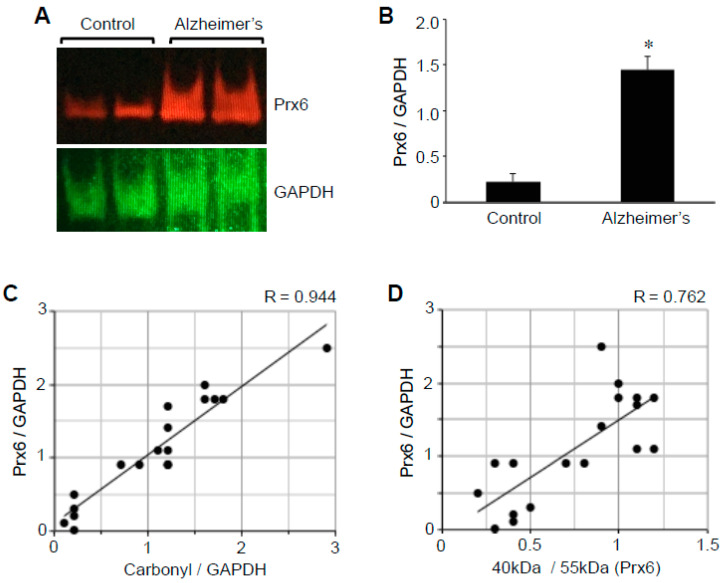
The Prx6 protein expression is upregulated in the brains of patients with Alzheimer’s disease. Brain (hippocampus) homogenates from control subjects and patients with Alzheimer’s disease were subjected to Western blotting using antibodies against Prx6 and GAPDH. (**A**) Representative results. (**B**) The bar graph represents means ± SEM of the ratio of Prx6 to GAPDH. The symbol * denotes that the value is significantly different from the control value at *p* < 0.05. (**C**) The scattered graph represents protein carbonylation vs. Prx6 expression (N = 18). Pearson correlation coefficient R = 0.944 (strong positive correlation). (**D**) The scattered graph represents Prx6 thiol oxidation vs. Prx6 expression (N = 18). Pearson correlation coefficient R = 0.762 (strong positive correlation).

We also found that the expression level of the Prx6 protein is higher in the brain of patients with Alzheimer’s disease compared with those of controls (Figure 5A), which may reflect the activation of an antioxidant defense mechanism in response to oxidative stress. The use of GAPDH protein as a loading control and the densitometry analysis showed that the protein expression levels of Prx6 between Alzheimer’s and control brains are significantly (6.5-fold) different (Figure 5B). The range of the Prx6 to GAPDH ratio determined in the brain of patients with Alzheimer’s was from 0.9 to 2.5 (mean = 1.4 ± 0.1), while in the brains of controls, it was from 0.01 to 0.5 (mean = 0.2 ± 0.08). The Prx6 protein expression was found to correlate strongly with protein carbonylation (Figure 5C), as well as the Prx6 thiol oxidation as measured by the ratio of 40 to 55 kDa species (Figure 5D).

The analysis of Aβ plaque and neuritic plaque scores in relation to hippocampal brain Prx6 levels may have shown a tendency to be dependent on the severity of Alzheimer’s disease, but no statistically significant differences were noted among patients’ brain tissues. The Prx6 content was found to be consistently higher with statistical significance between controls and patients with Alzheimer’s of any degree of severity.

### 2.5. Correlation Analysis to Examine the Relationships between ACE2 and Oxidative Stress

**Figure 6:**
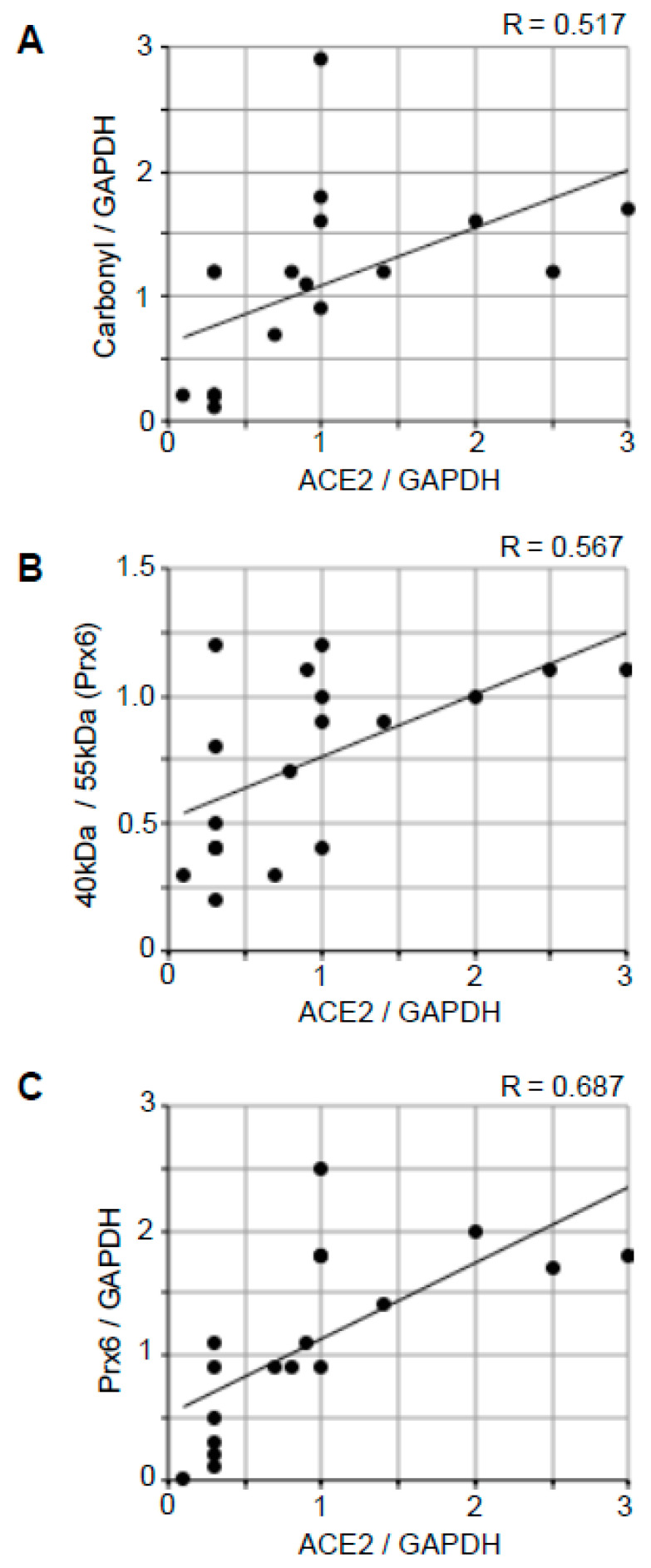
Correlation analysis to examine the relationship between ACE2 protein expression and oxidative stress. The scattered graphs represent (**A**) ACE2 expression vs. protein carbonylation, (**B**) ACE2 expression vs. Prx6 thiol oxidation, and (**C**) ACE2 expression vs. Prx6 expression using Western blotting data (N = 18). Pearson correlation coefficients indicate moderate positive correlations.

Figure 6 shows the results of correlation analyses to examine the relationships between ACE2 and various oxidative stress parameters using all 18 samples (13 patients with Alzheimer’s disease and five controls). The Pearson correlation coefficient R-value between the ACE2 expression and the protein carbonyl content was found to be 0.517 (Figure 6A), and the R-value between the ACE2 expression and the Prx6 thiol oxidation state was 0.567 (Figure 6B). The R-value between the ACE2 expression and the Prx6 expression was found to be slightly higher with 0.687 (Figure 6C). Thus, the ACE2 protein expression has a moderate positive correlation with oxidative stress parameters and the upregulation of an antioxidant.

## 3. Discussion

The major physiological function of ACE2 is to lower blood pressure by catalyzing the hydrolysis of angiotensin II, which acts as a vasoconstrictor, into angiotensin (1–7) that functions as a vasodilator [32]. ACE2 also serves as the receptor for SARS-CoV-2, the virus that is causing the current COVID-19 pandemic. Alveolar epithelial cells and cells of the conducting airways are the primary targets of SARS-CoV-2, resulting in severe pneumonia and acute respiratory distress syndrome [33]. In the brain, immunohistochemistry detected the ACE2 protein expression in endothelial and arterial smooth muscle cells [34]. In addition, cultured brain glial cells [35] and mouse brain neurons [36] have been shown to express ACE2 [37].

The major finding of the present study is that the brain ACE2 expression is higher in patients with Alzheimer’s disease than in controls. We did not detect a correlation between the severity of Alzheimer’s disease and the ACE2 expression, and the ACE2 was found to be upregulated even in mild cases. These results indicate that the infection of patients with Alzheimer’s disease of any disease severity with SARS-CoV-2 results in a higher viral entry into brain cells. Thus, patients with Alzheimer’s disease are at risk of being highly affected by COVID-19. No gender difference was noted in the ACE2 protein expression in brains with Alzheimer’s, and the ACE2 protein expression was not dependent on age. As such, it appears that there is a direct relationship between Alzheimer’s disease and the ACE2 expression. Understanding the mechanism of this relationship may shed light on the pathogenesis of Alzheimer’s disease, as well as possible management strategies for individuals infected with SARS-CoV-2, as ACE2 is the host cell receptor for this virus.

Oxidative stress has been implicated in Alzheimer’s disease and there have been many studies monitoring various oxidative stress parameters [7,8,9,10]. Thus, redox processes may play a role in regulating the ACE2 gene expression. Consistent with the previous study [38], DNPH-reactive protein carbonyls were found to be increased in our Alzheimer’s disease brain samples.

Information about protein thiol oxidation in human brains with Alzheimer’s has been limited. The thiol redox status of proteins plays pivotal roles in biology [39,40,41,42]. As proteins are the functional molecules in the biological system, their redox status directly reflects pathophysiology. While there are a number of ways to monitor the oxidation of sulfhydryl groups in the biological samples, most of the techniques only allow for the global assessment of oxidation in cell or tissue samples [43], and the monitoring of the redox status of specific proteins cannot be readily performed. Some techniques can identify individual proteins that are oxidized, but these approaches are difficult, time-consuming, and expensive [44,45]. The -*SulfoBiotics*- Protein Redox State Monitoring Kit Plus is remarkable in that it can readily allow for the assessment of the redox states of specific proteins of interest in the biological samples in a convenient and cost-effective fashion [31]. To understand the thiol biology, we previously performed a redox state monitoring analysis of Prx6 using the -*SulfoBiotics*- Protein Redox State Monitoring Kit Plus system [31]. In this system, a 15 kDa Protein-SHifter is added to every reduced cysteine residue, defining protein thiol redox states while examining the mobility shift caused by the Protein-SHifters using gel electrophoresis. Thiol status specifically on Prx6 was subsequently determined by Western blotting. In humans, the Prx6 protein molecule contains one catalytic cysteine and one additional noncatalytic cysteine. We found that the treatment of cultured cells with hydrogen peroxide caused the oxidation of the catalytic cysteine with minimal influence on the noncatalytic cysteine [31]. In the brains of patients with Alzheimer’s disease, the present study also showed that the catalytic cysteine is preferentially oxidized without significant influence on the noncatalytic cysteine of Prx6. The protein thiol oxidation through the measurement of the ratio of 40 to 55 kDa species of Prx6 correlated with the degree of protein carbonylation.

Peroxiredoxins are a class of peroxidase antioxidant enzymes [46,47]. Six members of peroxiredoxins have been identified [46,47,48,49], with Prx1 to Prx5 containing two catalytic cysteines that perform the two-electron reduction of hydrogen peroxide to water [46,47]. Prx6, however, is a unique peroxiredoxin that possesses only one catalytic cysteine and presumably utilizes glutathione to receive the second electron for the two-electron reduction [50,51]. The present study also found that the Prx6 protein expression is higher in the brains of patients with Alzheimer’s disease, supporting the concept that these brains have been adapted to contain increased antioxidant defenses. Similar to our results on Prx6, it has been reported that the protein expression levels of Prx1 and Prx2 in the brain are also increased in Alzheimer’s disease [52]. In our study, the increased Prx6 protein expression was strongly correlated with the degree of oxidative stress as measured by protein carbonylation and Prx6 thiol oxidation.

The correlation analysis of the brain ACE2 protein expression and various oxidative stress parameters, including protein carbonylation, cysteine oxidation of Prx6, and Prx6 protein expression, showed positive relationships. The relationship between ACE2 and Prx6 was found to be highest. However, the correlations between these redox parameters and the ACE2 expression were found to be modest, suggesting other factors may also participate in regulating ACE2 gene expression in Alzheimer’s disease. Ascertaining whether the mechanism of the upregulation of ACE2 expression involves oxidative stress and whether increased ACE2 promotes oxidative stress requires further investigation.

## 4. Materials and Methods

### 4.1. Patient Samples

Frozen brain (hippocampus) tissues from de-identified deceased patients who had been diagnosed with Alzheimer’s disease and those from control individuals without Alzheimer’s or other neurological diseases were obtained from the Georgetown University Medical Center Brain Bank in Washington, DC. Formalin-fixed and paraffin-embedded hippocampus tissues from the same patients were also obtained. The patients usually died at home and full autopsies were not performed in most of these cases. The bodies of deceased patients were maintained in refrigeration until tissue collection could occur. The mean postmortem interval value for patients with Alzheimer’s disease was 13.8 ± 1.9 h and that of controls was 15.2 ± 2.5 h. After brain removal, tissues were flash-frozen on dry ice and then stored at −80 °C. Control brain tissues were obtained using the same protocols. ABC grading for Alzheimer’s disease was performed histologically as previously described [24], at the time of banking by Brent Harris, a neuropathologist and co-author of this paper.

### 4.2. Tissue Homogenate Preparations

Frozen hippocampus brain tissues were homogenized in a 4 volume of the 10% trichloroacetic acid (VWR International, Radnor, PA, USA) solution using a Kontes glass tissue grinder. Homogenized tissues were incubated on ice for 30 min and centrifuged for 4 min at 8000 rpm at 4 °C in an accuSpin Micro R centrifuge (ThermoFisher Scientific, Waltham, MA, USA). The resultant pellets were then washed with acetone three times, then with ethanol once. Pellets were air-dried, resuspended in the Lysate Buffer (supplied in the -*SulfoBiotics*- Protein Redox State Monitoring Kit Plus, Dojindo Molecular Technologies Inc., Rockville, MD, USA), supplemented with phenylmethylsulfonyl fluoride (2 mM), leupeptin (10 µg/mL), and aprotinin (10 µg/mL) (MilliporeSigma, Burlington, MA, USA), and sonicated on ice using an ultrasonic processor. Samples were then centrifuged at 13,000 rpm for 10 min, and the supernatants were collected. Protein concentrations of cell lysates were measured using the Pierce bicinchoninic acid assay (Thermo Fisher Scientific, Waltham, MA, USA).

### 4.3. Western Blotting

Equal amounts of protein samples (5 µg) were electrophoresed through a reducing sodium dodecyl sulfate polyacrylamide gel. Proteins were then electro-transferred to the Immobilon-FL Transfer Membrane (MilliporeSigma, Burlington, MA, USA). The membranes were blocked with Odyssey blocking buffer (LI-COR, Lincoln, NE, USA) for one hour at 25 °C and incubated overnight with the rabbit ACE2 antibody (Catalog # 4355; Cell Signaling), the goat anti-GAPDH antibody (Catalog # PLA0302; MilliporeSigma), or the rabbit anti-Prx6 antibody (Catalog # P0058; MilliporeSigma) at 4 °C. Washed membranes were then incubated with IRDye 680RD or IRDye 800CW (LI-COR) for one hour at 25 °C in the dark. Signals were obtained using the Odyssey Infrared Imaging System (LI-COR), and Western blot band intensities were analyzed by densitometry.

### 4.4. Measurements of Protein Carbonylation

Carbonyl groups in protein side chains were derivatized with DNPH [53] to form the 2,4-dinitrophenyl (DNP) hydrazone derivative for detection using the OxyBlot Protein Oxidation Detection Kit (MilliporeSigma), according to the manufacturer’s instructions for use with rabbit anti-DNP antibody at 1:150 dilution for Western blotting. The Immobilon-FL Transfer Membrane was then incubated with anti-rabbit IRDye 680RD for one hour. Afterward, signals were captured using the Odyssey Infrared Imaging System, and band intensities were analyzed by densitometry.

### 4.5. Protein Thiol Redox State Monitoring

Protein thiol redox states were monitored using the -*SulfoBiotics*- Protein Redox State Monitoring Kit Plus (Catalog # SB12; Dojindo Molecular Technologies). Samples were labeled with the Protein-SHifter Plus in accordance with the manufacturer’s instructions. After cell extracts were subjected to SDS-PAGE, gels were exposed to UV light on a transilluminator to remove Protein-SHifter. Proteins in the gel were then electro-transferred to the Immobilon-FL Transfer Membrane. The membrane was blocked with Odyssey Blocking Buffer for one hour at 25 °C and incubated overnight with the rabbit anti-Prx6 antibody (Catalog # P0058; MilliporeSigma) at 4 °C. Washed membranes were then incubated with anti-rabbit IRDye 680RD for one hour at 25 °C in the dark. Afterward, signals were then captured by using the Odyssey Infrared Imaging System, and band intensities were analyzed by densitometry.

### 4.6. Statistical Analysis

Means and standard errors of mean (SEM) were computed. Two groups were compared using a two-tailed Student’s *t* test, and differences between more than two groups were determined by the analysis of variance (ANOVA). *p* < 0.05 was defined to be statistically significant.

## 5. Conclusions

In summary, the examination of brains from patients with Alzheimer’s disease indicated that ACE2 protein is upregulated in association with oxidative stress. Further work is needed to determine whether redox processes participate in the mechanism of ACE2 gene expression or whether ACE2 regulates oxidative stress in the brain. We analyzed 13 patients with Alzheimer’s disease and five controls. While the number of the latter is relatively small, observed values for these individuals were very consistent in all the parameters measured in the present study. In addition, in our study, the mean age was higher for patients with Alzheimer’s disease than for controls, yet the ACE2 protein expression was found not to be dependent on age, as no correlation was detected (with a very low Pearson correlation coefficient of 0.059). Thus, it is not likely that these limitations dampen our conclusion that Alzheimer’s disease per se is associated with increased ACE2 protein expression. Our conclusion is further supported by a communication by Lim et al. [54] published as a Letter to the Editor that their genome-wide association study detected upregulated *Ace2* mRNA in the hippocampus of patients with Alzheimer’s disease. By contrast, Kehoe et al. [55] reported that ACE2 peptidase enzyme activity was reduced in such patients. Our results are the first to report the levels of ACE2 protein expression in Alzheimer’s. In light of the importance of ACE2 protein in the current pandemic as the SARS-CoV-2 receptor, further investigations on the issue of ACE2 in brains with Alzheimer’s disease are warranted, considering the possibility that the high level of ACE2 in patients with Alzheimer’s may affect their responses to COVID-19.

## Data Availability

The data presented in this study are available on request from the corresponding author.

## Abbreviations

ACE2	angiotensin-converting enzyme 2
COVID-19	coronavirus disease 2019
DNP	2,4-dinitrophenyl
DNPH	2,4-dinitrophenylhydrazine
GAPDH	glyceraldehyde 3-phosphate dehydrogenase
Prx6	peroxiredoxin 6
ROS	reactive oxygen species
SARS-CoV-2	Severe acute respiratory syndrome coronavirus 2
SEM	standard errors of mean

## References

[1] Masters C.L., Bateman R., Blennow K., Rowe C.C., Sperling R.A., Cummings J.L. (2015). Alzheimer’s disease. Nat. Rev. Dis. Primers..

[2] Scheltens P., Blennow K., Breteler M.M., de Strooper B., Frisoni G.B., Salloway S., Van der Flier W.M. (2016). Alzheimer’s disease. Lancet.

[3] Ballard C., Gauthier S., Corbett A., Brayne C., Aarsland D., Jones E. (2011). Alzheimer’s disease. Lancet.

[4] Querfurth H.W., LaFerla F.M. (2010). Alzheimer’s disease. N. Engl. J. Med..

[5] Freeman B.A., Crapo J.D. (1982). Biology of disease: Free radicals and tissue injury. Lab. Investig..

[6] Halliwell B., Gutteridge J. (2007). Free Radicals in Biology and Medicine.

[7] Tabner B.J., El-Agnaf O.M., Turnbull S., German M.J., Paleologou K.E., Hayashi Y., Cooper L.J., Fullwood N.J., Allsop D. (2005). Hydrogen peroxide is generated during the very early stages of aggregation of the amyloid peptides implicated in Alzheimer disease and familial British dementia. J. Biol. Chem..

[8] Butterfield D.A., Perluigi M., Sultana R. (2006). Oxidative stress in Alzheimer’s disease brain: New insights from redox proteomics. Eur. J. Pharmacol..

[9] Zhao Y., Zhao B. (2013). Oxidative stress and the pathogenesis of Alzheimer’s disease. Oxid. Med. Cell. Longev..

[10] Chen Z., Zhong C. (2014). Oxidative stress in Alzheimer’s disease. Neurosci. Bull..

[11] Polidori M.C., Nelles G. (2014). Antioxidant clinical trials in mild cognitive impairment and Alzheimer’s disease—challenges and perspectives. Curr. Pharm. Des..

[12] Persson T., Popescu B.O., Cedazo-Minguez A. (2014). Oxidative stress in Alzheimer’s disease: Why did antioxidant therapy fail?. Oxid. Med. Cell. Longev..

[13] Su S., Wong G., Shi W., Liu J., Lai A.C.K., Zhou J., Liu W., Bi Y., Gao G.F. (2016). Epidemiology, genetic recombination, and pathogenesis of coronaviruses. Trends Microbiol..

[14] Satija N., Lal S.K. (2007). The molecular biology of SARS coronavirus. Ann. N. Y. Acad. Sci..

[15] Wu F., Zhao S., Yu B., Chen Y.M., Wang W., Song Z.G., Hu Y., Tao Z.W., Tian J.H., Pei Y.Y. (2020). A new coronavirus associated with human respiratory disease in China. Nature.

[16] Huang C., Wang Y., Li X., Ren L., Zhao J., Hu Y., Zhang L., Fan G., Xu J., Gu X. (2020). Clinical features of patients infected with 2019 novel coronavirus in Wuhan, China. Lancet.

[17] Yan R., Zhang Y., Li Y., Xia L., Guo Y., Zhou Q. (2020). Structural basis for the recognition of the SARS-CoV-2 by full-length human ACE2. Science.

[18] Tai W., He L., Zhang X., Pu J., Voronin D., Jiang S., Zhou Y., Du L. (2020). Characterization of the receptor-binding domain (RBD) of 2019 novel coronavirus: Implication for development of RBD protein as a viral attachment inhibitor and vaccine. Cell. Mol. Immunol..

[19] Yang J., Zheng Y., Gou X., Pu K., Chen Z., Guo Q., Ji R., Wang H., Wang Y., Zhou Y. (2020). Prevalence of comorbidities and its effects in patients infected with SARS-CoV-2: A systematic review and meta-analysis. Int. J. Infect. Dis..

[20] Li B., Yang J., Zhao F., Zhi L., Wang X., Liu L., Bi Z., Zhao Y. (2020). Prevalence and impact of cardiovascular metabolic diseases on COVID-19 in China. Clin. Res. Cardiol..

[21] Mao L., Jin H., Wang M., Hu Y., Chen S., He Q., Chang J., Hong C., Zhou Y., Wang D. (2020). Neurological manifestations of hospitalized patients with Coronavirus Disease 2019 in Wuhan, China. JAMA Neurol..

[22] Anand K.S., Dhikav V. (2012). Hippocampus in health and disease: An overview. Ann. Indian Acad. Neurol..

[23] Setti S.E., Hunsberger H.C., Reed M.N. (2017). Alterations in hippocampal activity and Alzheimer’s disease. Transl. Issues Psychol. Sci..

[24] Hyman B.T., Phelps C.H., Beach T.G., Bigio E.H., Cairns N.J., Carrillo M.C., Dickson D.W., Duyckaerts C., Frosch M.P., Masliah E. (2012). National Institute on Aging-Alzheimer’s Association guidelines for the neuropathologic assessment of Alzheimer’s disease. Alzheimers Dement..

[25] Hedreen J.C., Raskin L.S., Price D.L. (1994). A quick silver method for senile plaques and neurofibrillary tangles in paraffin sections. Brain Res. Bull..

[26] Reusche E. (1991). Silver staining of senile plaques and neurofibrillary tangles in paraffin sections. A simple and effective method. Pathol. Res. Pract..

[27] DeTure M.A., Dickson D.W. (2019). The neuropathological diagnosis of Alzheimer’s disease. Mol. Neurodegener..

[28] Ryan N.S., Rossor M.N., Fox N.C. (2015). Alzheimer’s disease in the 100 years since Alzheimer’s death. Brain.

[29] Serrano-Pozo A., Frosch M.P., Masliah E., Hyman B.T. (2011). Neuropathological alterations in Alzheimer disease. Cold Spring Harb. Perspect. Med..

[30] Goedert M., Jakes R., Vanmechelen E. (1995). Monoclonal antibody AT8 recognizes tau protein phosphorylated at both serine 202 and threonine 205. Neurosci. Lett..

[31] Suzuki Y.J., Marcocci L., Shimomura T., Tatenaka Y., Ohuchi Y., Brelidze T.I. (2019). Protein redox state monitoring studies of thiol reactivity. Antioxidants.

[32] Gheblawi M., Wang K., Viveiros A., Nguyen Q., Zhong J., Turner A.J., Raizada M.K., Grant M.B., Oudit G.Y. (2020). Angiotensin-converting enzyme 2: SARS-CoV-2 receptor and regulator of the renin-angiotensin system: Celebrating the 20th anniversary of the discovery of ACE2. Circ. Res..

[33] Xu Z., Shi L., Wang Y., Zhang J., Huang L., Zhang C., Liu S., Zhao P., Liu H., Zhu L. (2020). Pathological findings of COVID-19 associated with acute respiratory distress syndrome. Lancet Respir. Med..

[34] Hamming I., Timens W., Bulthuis M.L.C., Lely A.T., Navis G.J., Van Goor H. (2004). Tissue distribution of ACE2 protein, the functional receptor for SARS coronavirus. A first step in understanding SARS pathogenesis. J. Pathol..

[35] Gallagher P.E., Chappell M.C., Ferrario C.M., Tallant E.A. (2006). Distinct roles for ANG II and ANG-(1–7) in the regulation of angiotensin-converting enzyme 2 in rat astrocytes. Am. J. Physiol. Cell Physiol..

[36] Doobay M.F., Talman L.S., Obr T.D., Tian X., Davisson R.L., Lazartigues E. (2007). Differential expression of neuronal ACE2 in transgenic mice with overexpression of the brain renin-angiotensin system. Am. J. Physiol. Regul. Integr. Comp. Physiol..

[37] Xia H., Lazartigues E. (2008). Angiotensin-converting enzyme 2 in the brain: Properties and future directions. J. Neurochem..

[38] Aksenov M.Y., Aksenova M.V., Butterfield D.A., Geddes J.W., Markesbery W.R. (2001). Protein oxidation in the brain in Alzheimer’s disease. Neuroscience.

[39] Boronat S., Domènech A., Hidalgo E. (2017). Proteomic characterization of reversible thiol oxidations in proteomes and proteins. Antioxid. Redox Signal..

[40] Chung H.S., Wang S.B., Venkatraman V., Murray C.I., Van Eyk J.E. (2013). Cysteine oxidative posttranslational modifications: Emerging regulation in the cardiovascular system. Circ. Res..

[41] Sen C.K. (2000). Cellular thiols and redox-regulated signal transduction. Curr. Top. Cell. Regul..

[42] Suzuki Y.J., Forman H.J., Sevanian A. (1997). Oxidants as stimulators of signal transduction. Free Radic. Biol. Med..

[43] Rudyk O., Eaton P. (2014). Biochemical methods for monitoring protein thiol redox states in biological systems. Redox Biol..

[44] Leichert L.I., Gehrke F., Gudiseva H.V., Blackwell T., Ilbert M., Walker A.K., Strahler J.R., Andrews P.C., Jakob U. (2008). Quantifying changes in the thiol redox proteome upon oxidative stress in vivo. Proc. Natl. Acad. Sci. USA.

[45] Couvertier S.M., Zhou Y., Weerapana E. (2014). Chemical-proteomic strategies to investigate cysteine posttranslational modifications. Biochim. Biophys. Acta.

[46] Rhee S.G. (2016). Overview on peroxiredoxin. Mol. Cells.

[47] Rhee S.G., Kil I.S. (2017). Multiple functions and regulation of mammalian peroxiredoxins. Annu. Rev. Biochem..

[48] Seo M.S., Kang S.W., Kim K., Baines I.C., Lee T.H., Rhee S.G. (2000). Identification of a new type of mammalian peroxiredoxin that forms an intramolecular disulfide as a reaction intermediate. J. Biol. Chem..

[49] Kim K., Kim I.H., Lee K.Y., Rhee S.G., Stadtman E.R. (1988). The isolation and purification of a specific ‘protector’ protein which inhibits enzyme inactivation by a thiol/Fe(III)/O2 mixed-function oxidation system. J. Biol. Chem..

[50] Fisher A.B. (2011). Peroxiredoxin 6: A bifunctional enzyme with glutathione peroxidase and phospholipase A2 activities. Antioxid. Redox Signal..

[51] Fisher A.B. (2017). Peroxiredoxin 6 in the repair of peroxidized cell membranes and cell signaling. Arch. Biochem. Biophys..

[52] Kim S.H., Fountoulakis M., Cairns N., Lubec G. (2001). Protein levels of human peroxiredoxin subtypes in brains of patients with Alzheimer’s disease and Down syndrome. J. Neural Transm. Suppl..

[53] Levine R.L., Williams J.A., Stadtman E.R., Shacter E. (1994). Carbonyl assays for determination of oxidatively modified proteins. Methods Enzymol..

[54] Lim K.-H., Yang S., Kim S.-H., Joo J.-Y. (2020). Elevation of ACE2 as a SARS-CoV-2 entry receptor gene expression in Alzheimer’s disease (Letter to the Editor). J. Infect..

[55] Kehoe P.G., Wong S., Mulhim N.A., Palmer L.E., Miners J.S. (2016). Angiotensin-converting enzyme 2 is reduced in Alzheimer’s disease in association with increasing amyloid-β and tau pathology. Alzheimers Res. Ther..

